# Tuberculosis infection control practices and associated factors among health care workers in health centers of West Gojjam zone, Northwest Ethiopia: a cross-sectional study

**DOI:** 10.1186/s12913-016-1608-y

**Published:** 2016-08-08

**Authors:** Kassahun Tamir, Belaynew Wasie, Muluken Azage

**Affiliations:** 1 MNCH Case Team, Health Promotion and Disease Prevention Process, Amhara Regional Health Bureau, P.O. Box: 495, Bahir Dar, Ethiopia; 2 School of Public Health, College of Medicine and Health Sciences, Bahir Dar University, Bahir Dar, Ethiopia

**Keywords:** Tuberculosis, Infection control practices, Health care workers, Health centers, Ethiopia

## Abstract

**Background:**

Tuberculosis (TB) remains a major global health problem. The emerging epidemic of multi- and extensively drug-resistant (M/XDR) TB further imperils health workers, patients and public health. Health facilities with inadequate infection control are risky environments for the emergence and transmission of TB. There was no study that presented data on infection control practices of health care workers. This study aimed to assess tuberculosis infection control practices and associated factors among health care workers in West Gojjam Zone, Northwest Ethiopia.

**Methods:**

Institution based quantitative cross-sectional study triangulated with qualitative observation and key informant interview was conducted. Six hundred sixty two health care workers were selected by multistage random sampling method. Self-administered structured questionnaire was used to collect quantitative data. Observation checklists and key informant interview guides were used to collect qualitative data. Quantitative data were entered in to Epi Info version 3.5.3 and analyzed using SPSS version 20. Odds ratio with 95 % confidence interval was used to identify factors associated with TB infection control practice of health care workers. Qualitative data were translated, transcribed, analyzed and triangulated with the quantitative findings.

**Results:**

The proportion of proper TB infection control (TBIC) practices was 38 %. Qualitative data showed that administrative, environmental and personal respiratory protection control measures were not practiced well. Knowledge on the presence of TBIC plan [AOR = 4.25, 95 % CI: 2.46 - 7.35], knowledge on the presence of national guideline [AOR = 8.95, 95 % CI: 4.35 - 18.40] and working department of the health care workers were independent predictors of TBIC practices.

**Conclusions:**

The proportion of proper TBIC practices of health care workers was low. TBIC practices were determined by knowing the presence of TBIC plan and national guideline and working department. Hence, supportive supervision and trainings should be given to health care workers who are working other than TB clinics to improve the knowledge of TBIC plan and guidelines. Health centers shall prepare TBIC plans and orient all health care workers.

## Background

Tuberculosis (TB) remains a major global health problem. It causes ill-health among millions of people each year and ranks as the second leading cause of death from an infectious disease worldwide, after the Human Immunodeficiency Virus (HIV). The number of TB deaths was large given that most are preventable if people can access health care for a diagnosis and the right treatment is provided [1].

TB has been declared as a global emergency since 1993 and there is globally recommended TB control strategy. The DOTS (Directly Observed Treatment, Short-Course) strategy was launched in 1994 by the World Health Organization (WHO) to ensure that infectious TB patients are identified and cured using a standardized drug combination. Later to build on the achievements of DOTS and address the remaining challenges, the stop TB strategy was launched by WHO in 2006 to help achieve the millennium development goals for TB in 2015. The Strategy has six components where DOTS remains the most important component of the strategy. The components are pursuing high quality DOTS expansion and enhancement; address TB/HIV, MDR-TB and the needs of poor and vulnerable population, contribute to health system strengthening based on primary health care, engage all care providers, empower people with TB and communities through partnership and enable and promote research [2].

Ethiopia is one of the countries among the 22 high TB burdened countries and the 27 high MDR TB burden countries in the world. Compounded with HIV/AIDS, TB has become a formidable threat to the country [3]. The emerging epidemic of M/XDR TB further endangers patients and public health [4]. It remains a very important occupational risk for health care workers (HCWs) in low- and middle- income countries (LMICs) and for workers in some institutions in high income countries (HICs). Risk appears particularly high when there is increased exposure combined with inadequate infection control measures [5–10].

A study conducted on the risk of tuberculosis infection and disease associated with work in health care settings of LMICs showed that the median prevalence of latent TB infection (LTBI) was 63 % (range 33–79 %) [11]. A systematic review on TB among HCWs in LMICs showed that the prevalence of LTBI among HCWs was 54 % (range 33 to 79 %). Estimates of the annual risk of LTBI ranged from 0.5 to 14.3 %, and the annual incidence of TB disease in HCWs ranged from 69 to 5,780 per 100,000 [12]. Higher rate of M/XDR-TB among HCWs compared with the general population leads to severe medical and psychosocial consequences [4].

Global TB and HIV experts advocate infection control as a key TB control strategy [4, 13]. Components to good work practice on TBIC include an infection control plan; administrative support to implement the plan; training of staff; education of patients and increasing community awareness; coordination between the HIV and TB programs; ensuring environmental controls including ventilation (natural and mechanical), room air cleaners (generally filters or ultra violet germicidal irradiation [UVGI]), or UVGI in the upper room, and using respiratory protection [6, 9, 14, 15].

There were no enough studies conducted to assess level of TBIC practices and associated factors among HCWs in Ethiopia particularly in health center settings. Therefore, the current study was conducted to determine level of practice on TBIC and its associated factors among HCWs.

## Methods

### Setting

The study was conducted in the West Gojjam Zone, Northwest Ethiopia. The Zone has 2,382,497 (50.4 % male and 49.6 % female) population, from which 2,134,666 (87 %) reside in rural and 247,831 (13 %) live in urban areas. There are 18 Woredas (the administrative level next to Zone), 13 of them are rural and 5 are urban. It has 394 kebeles (the lowest administrative level) (363 rural and 31 urban). There were one general government hospital and 91 health centers. Above 3500 HCWs deployed in ART clinic, TB clinic, laboratory, pharmacy, outpatient/inpatient and mother and child health departments of these health facilities during the time of the study [16].

### Study design

A mixed methods study using institution based quantitative cross-sectional study design triangulated with qualitative interview and observation was conducted to assess TBIC practices and associated factors among HCWs from June to September 2014.

### Participants

All HCWs working in all health centers of West Gojjam zone were the study population. All health professionals who were providing health care services were eligible for the study.

#### Sample size and technique

Sample size was computed using Epi Info version 3. 5.3 by considering the proportion (p) of good TB infection control practices (using respiratory protection) 54.7 % from a previous study [4], a design effect of 2 for multi stage sampling and a margin of error of 5 %. The final sample size was 662.

**Fig. 1:**
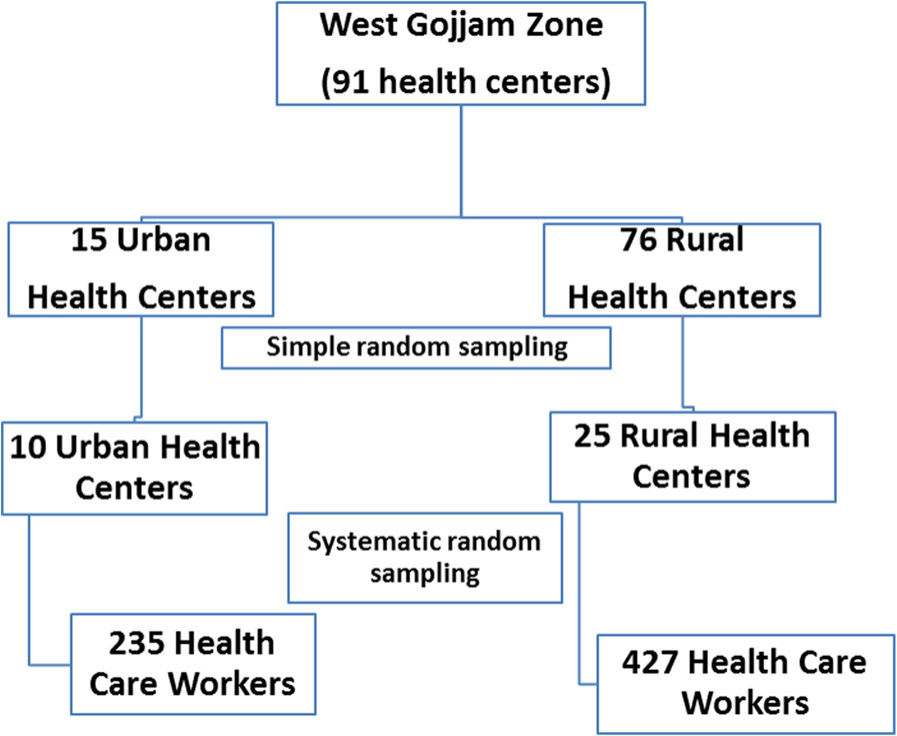
Schematic presentation of sampling procedure for the quantitative study

A multistage sampling technique was used. In the first stage, 35 health centers (10 urban and 25 rural), according to the number of health centers in each category, were selected using simple random sampling technique from 91 health centers. The number of health centers included in the sample was dependent on inclusion of adequate number of at least 20 % of facilities. In stage two, study participants were selected using systematic random sampling technique. Proportion to size allocation was used to determine the required study participants in randomly selected health centers (Fig. 1). A total of 15 health centers were included for observation of TBIC practices. A total of 15 key informants, one from each selected health center, who were heads of the selected health centers were selected using a criterion type of purposive sampling method for qualitative data to explore detailed information on the actual TBIC practices and overall settings.

### Study variables

The dependent variable of the study was the level of practice on TBIC (rated as proper or improper). The practices related to TBIC were separate cases/suspects, give priority for coughing patients, screen patients for TB, give health education about cough hygiene, staff check-up of own HIV status every three months, staff seek own TB diagnosis if symptomatic, staff redeployment from high risk to low risk working departments if hypothetically HIV positive, open doors and windows in working area for natural ventilation, using personal protective equipment (respirators for staff) and using/referring the national guideline for daily TBIC activities. Independent variables were socio-demographic variables (age, sex, work experience, profession, and level of education), training, working department, presence of resources; type of construction of rooms, knowledge on presence of TBIC plan and national guideline, awareness on presence of national guideline, lack of TBIC focal person, perceived barriers on TBIC activities. The overall TBIC practices were assessed by asking above ten questions and 80 % overall practice score was used as a cut-off point to determine proper TBIC practice level. Participants who scored less than 80 % were considered as having improper TBIC practice and those who scored greater than or equal to 80 % were considered as having proper TBIC practice.

### Data collection

Data were collected using self-administered structured questionnaire. Pre-test was done in other study settings to evaluate face validity and modifications of some questions were made. The questionnaire was prepared first in English and then translated to Amharic language and then again back translated to English by a different public health expert. The questionnaire included questions about the participant’s socio-demographic characteristics, work place practice and administrative controls, environmental controls and personal respiratory protection practices and questions related to access to voluntary counseling and testing (VCT) and TB/ HIV care.

Qualitative data were collected from 15 health centers using observation and key informant interviews until the obtained information were saturated. The data were transcribed, analyzed and triangulated with the quantitative data findings. It was used to explain the result of quantitative study. Six diploma nurses who know the topography of West Gojjam Zone were recruited, trained and collected the data. One BSc nurse supervised the data collection process.

### Data analysis

Quantitative data were checked for completeness and entered in to Epi Info version 3.5.3 statistical package and exported to SPSS version 20 for analysis. Mean, standard deviation, and percentages were used to describe data. Odds Ratio with 95 % CI, to ascertain the association between dependent and independent variables were calculated. Qualitative data analysis was started during data collection. The audio recordings of the interviews were transcribed and translated into English. The transcripts reflected verbatim (direct word by word) transcription of the recorded key informant interview. Each transcript was read multiple times by investigator. A coding scheme was developed based on the objectives of the study and the relevant themes that emerged from the transcripts. Thematic coding was performed on all transcripts using Open Code version 3.6.2.0 software. Quotes that represented the common themes were selected from the transcripts and included in the report.

### Ethical issues

The study was approved by research ethics committee of College of Medicine and Health Sciences, Bahir Dar University. A formal letter was given to Amhara Regional Health Bureau and then letter support was written to Zonal health office. West Gojjam Zonal health office, Woreda Health offices and head of health centers were communicated for permission and cooperation to conduct the survey. Verbal consent was taken from each study participants after briefing the study objective.

## Results

### Socio-demographic characteristics of participants

A total of 647 (with a response rate of 97.7 %) health care workers participated in the study. Those who didn’t participate were absent from work and some were incomplete questionnaires that were discarded due to loss of critical variable. Among these, 16 (2.4 %) questionnaires were incomplete and the response rate was 95.3 %. Four hundred ten (65 %) participants were working in rural health centers and 221 (35 %) were working in urban health centers.

Majority (61 %) of study participants were males. The mean (±SD) age of participants was 25.8 (±3.9) years. Two third (75.1 %) of the study participants had diploma. Above half (53.1 %) of the study participants were nurses, 12.8 % were health officers, 11.1 % were laboratory technologists, 10.9 % were midwives and 12 % were pharmacy by their profession. Nearly one third of the study participants (29.6 %) were working in adult outpatient department (Table 1).

**Table 1:** Socio-demographic characteristics of health care workers in West Gojjam Zone, Northwest Ethiopia, August 2014 (*n* = 631)

Variables	Number of health care workers	Percent
Site of health facility		
Rural	410	65.0
Urban	221	35.0
Sex		
Female	246	39.0
Male	385	61.0
Age (years)		
18–24	252	39.9
25–34	342	54.2
35–44	33	5.2
45–54	4	0.6
Educational level		
Degree	157	24.9
Diploma	474	75.1
Profession		
Health officer	81	12.8
Laboratory technologist	70	11.1
Midwifery	69	10.9
Nurse	335	53.1
Pharmacy	76	12
Working department		
Adult Outpatient	187	29.6
Antiretroviral therapy Clinic	30	4.8
Inpatient department	28	4.4
Laboratory department	70	11.1
Mother and Child	113	17.9
Pharmacy department	76	12.0
TB clinic	52	8.2
Under 5 clinic	75	11.9

### Characteristics of key informants and participants of observation

The age range of key informants was 20–29 years and eleven out of fifteen of them were males. Three out of fifteen key informants were diploma nurses and twelve were BSc holders. Ten out of fifteen key informants had an average of three years and above experience with their profession. The health facilities included in observation were 15 health centers. Within each health center TB clinic, IPD, adult OPDs, ART clinic, laboratory, pharmacy, mother and child departments were included for the observation.

### Tuberculosis infection control practices among health care workers

**Fig. 2:**
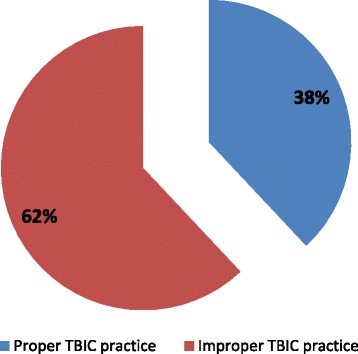
Tuberculosis infection control practice of health care workers, in West Gojjam Zone, Northwest Ethiopia, August 2014

The study showed that the overall proportion of proper TBIC practices was 38 %. Two hundred forty (38 %) participants scored greater than 80 % of the practice assessment but majority (62 %) of the health care workers had improper TBIC practices (Fig. 2).

TBIC practices were variable in different health centers studied. TBIC focal person was assigned in each health center; however, the presence of TBIC plan and national guideline for TBIC was known by 72.9 and 75.8 % of participants respectively. Above one third (34.5 %) of study participants were trained on TBIC and 71.6 % of participants reported that they were doing TB screening at their working department. Nearly a third (28.4 %) of study participants reported that TB cases or suspects were not routinely identified in their departments, and there was no separate waiting area for these patients. Nearly two-thirds (60 %) of study participants reported that they ‘always’ informed coughing patients about cough hygiene. More than half (54.8 %) of study participants reported that they were giving health education to TB patients and suspects.

**Fig. 3:**
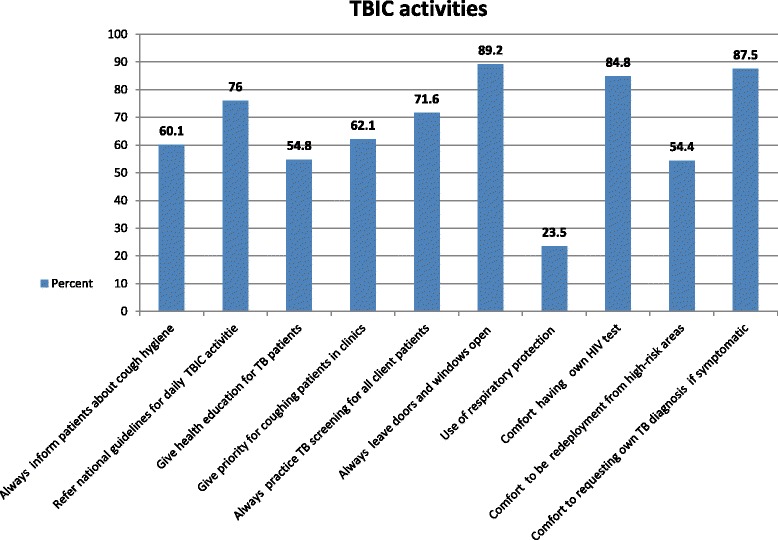
Tuberculosis infection control practices among health care workers, West Gojjam Zone, Northwest Ethiopia, August 2014

Three hundred seventy nine (60.1 %) HCWs reported that they always gave advice for coughing patients about cough hygiene. More than three quarters (76.0 %) of HCWs reported that they used the national guidelines for daily activities for TBIC. Nearly fifty five percent (54.8 %) of HCWs replied that they gave health education for TB patients. More than sixty percent (62.1 %) of HCWs reported that a prior health care service was given for coughing patients. More than one third of HCWs reported that they always practice TB screening techniques for all patients. Nearly ninety percent (89.1 %) of HCWs reported that they always leave doors and windows open during health care service provision. Nearly a quarter (23.5 %) of HCWs reported that they use a respirator in a room during health care provision for TB patients/suspects. Majority (84.8 %) of HCWs reported that they feel comfort with obtaining confidential own HIV testing for self at facility. More than half (54.4 %) of HCWs reported that they feel comfort with redeployment from high-risk areas if they are hypothetically HIV positive. Nearly ninety percent (87.5 %) of HCWs reported that they feel comfort with requesting own TB diagnosis for self if symptomatic (Fig. 3).

The qualitative study also showed that administrative TBIC activities were not implemented well. It was observed and interviewed that, even if there were TBIC plans developed by the TB focal persons and heads of the health centers it was not announced to all health care workers to act accordingly. “*This was due to the high work load on the health center and not giving priority. Even those health workers know the plans by any means are not doing such activities as planned”***(Health Care Worker 1)***.*

Separation of infectious patients was not practiced. Even MDR-TB patients were not separated in the clinic whilst they are awaiting transport to MDR-TB treatment facility. “*They are waiting within the community till they get the chance to treatment in the overburdened treatment site in Gondar hospital. The main reason is that there are no enough spaces and rooms to give care for separated TB cases so they get no separation care in our health center. Even the anti TB clinic is a room both outpatient examination and anti TB treatment services are given in because of the shortage of rooms in our health center”***(Health Care Worker 2)*.*** It was also interviewed only 1–2 health workers were trained on TB infection control practices in each health centers. “*Only two health workers were trained on TBIC in our health center and the majority of health workers don’t bother whether TBIC activities are practiced well according to the standards or not and they think that this is the assignment given to the two trained health workers working in the TB clinic”***(Health Care Worker 3)**.

TB cases and their families were educated on TB infection control practices. But it was observed that the health education given was irregular and partial. *“Health workers were not committed enough to give health education on TB and other cases with respecting their program scheduled. The health worker who is TBIC focal and working in TB clinic is considered the only responsible person to do this by other health workers. This is our big challenge in all health education programs not only in TBIC”***(Health Care Worker 1)**.

Many of participants (84.8 %) reported that they were comfortable to know their HIV status in their health facility and 54.4 % of who were comfortable with redeployment from high-risk areas if they are hypothetically HIV positive. Most of participants (87.5 %) were comfortable with requesting own TB diagnosis for self if symptomatic at their working health facility. Most participants (89.2 %) were always leaving doors and windows open in their working area to prevent the spread of TB. All of participants used natural ventilation and there were no other alternatives like room air cleaners and UVGI in the upper room to prevent the spread of TB.

The qualitative study also observed that only natural ventilations and open windows policy were used in the health facilities for TBIC. Even if windows and doors were there in majority of rooms it was not parallel and suitable for cross ventilation. Any other alternatives like propeller fans exhaust ventilation, high efficiency particulate air (HEPA) filters and UVGI Lights were not available in any of the rooms. Environmental controls were not periodically maintained with results written down in registers. It was also observed that there were no any areas designed to separate MDR-TB suspected or confirmed cases and there was no access to an engineer or other professional for assistance on design, installation, maintenance and assessment of environmental controls in each health centers.

Eighty one (12 %) participants reported that there were respirators in their working health facility and of these only 19(23 %) worn a respirator ‘always’ when they were entering in a room with a TB case/suspect. In the qualitative study it was also showed that personal respiratory protections were not practiced. In one urban health center it was stated that *“materials used for respiratory protection like N 95 s are not available in our health center most of the time. This is due to shortage of funds to purchase and avail it always. NGOs supporting us on this area are not bringing it for regular use. They bring it if there are MDR TB cases in the health center*” **(Health Care Worker 4)**.

It was showed that practice of various TBIC activities were differ among HCWs in West Gojjam Zone from using respiratory protection in a room with TB cases/suspects by only 19 (3 %) of participants to always leaving doors and windows open to prevent the spread of TB by 563 (89.2 %) of participants (Fig. 3).

### Reasons for not-practicing of TBIC among health workers

A number of challenges for the practicing of TBIC activities were raised in the qualitative part of the study. It was stated that structural barriers like lack of space to implement the separation of TB suspects, both out and in-patient settings were among those explored barriers. Although the HCWs knew that TB suspects should be separated, it was reported that this was not possible due to lack of space. Most of health centers reported that understaffing in their health facilities was a problem especially laboratory professionals. TBIC measures like screening for people with cough, health education and timely sputum examination were seen as additional tasks for the already overstretched staff. “*The laboratory professional in our health center was only one and he was busy to do the main investigations in our health center”***(Health Care Worker 5)***.*

Lack of managerial support including lack of funds to buy masks and to carry out renovations and structural improvements were also reported as challenges by many health centers. Lack of motivation and negative attitude towards TB among health workers and lack of adherence among patients was also among the challenges raised by many health centers. It was interviewed that, “*many of HCWs had negative attitudes towards TB work and they were not interested in TB related works and therefore TBIC control is left to the staff working in the TB clinics. Especially health workers working in pharmacy, MCH room, laboratory and inpatient departments were not giving attention to TBIC practice”***(Health Care Worker 1)***.*

The other challenge mentioned was a patient being non-compliant with cough hygiene*.* It was reported that where HCWs make an effort to implement the TBIC measures, patients don’t adhere to the instructions they are given. In the quantitative part participants reported that there were various barriers to implement TBIC practices. They cited patient non-compliance as a barrier to cough hygiene; patient complaints and occasional cold whether as barriers to natural ventilation; confidentiality and stigma concerns as barriers to staff testing themselves for TB/HIV as well as redeployment of HIV-positive HCWs; and insufficient supplies, discomfort and appearance as impediments to wider use of respirators by staff (Table 2).

**Table 2:** Reasons for not practicing TBIC among health care workers, West Gojjam Zone, August 2014

Variables	Frequency	Percent
Reasons for not informing about cough hygiene (*n* = 252)		
Patients won’t listen	118	46.8
Tissues and surgical masks are not available	70	27.8
Patients are not compliant	196	77.8
Health workers have no time to do so	85	33.7
Others	32	12.7
Reasons for not giving priority for coughing patients (*n* = 239)		
It is not fair	129	54
Complaint from other clients	210	87.9
Health workers don’t believe in prioritizing	59	24.7
Others	16	6.7
Reasons for not always opening windows/doors (*n* = 68)		
Patients complain when doors and windows are open	27	39.7
It is sometimes too cold	42	61.8
No one is in charge of this, responsibility diffusion	33	48.5
Others	11	16.2
Reasons for not using N95 respirators with TB cases/suspects(*n* = 62)		
No enough N95s	44	70.9
N95s are uncomfortable	20	32.3
Health workers unclear about use of N95s	28	45.2
N95s destroy their hairstyles	2	3.2
Lack of training on use of N95s	29	46.8
Others	10	16.1
Reasons for not testing for HIV(*n* = 96)		
Breach of confidentiality	77	80.2
Afraid of being tested	33	34.4
Do not want to know own HIV status	30	31.3
Possible stigma from other staff	65	67.7
Others	4	4.2
Reasons for not willing to be redeployment from high-risk units for HIV infected HCWs (*n* = 288)		
HCWs do not want to admit that they are HIV positive	107	37.2
Risk of disclosure of HIV status to colleagues	145	50.3
Colleagues may perceive them as avoidant	263	91.3
Others	37	12.8
Reasons for not comfortable with TB diagnosis (*n* = 79)		
Other staff link with HIV	41	51.9
Fear of rejection and stigma	49	62.0
Lack of trust on doctors to maintain confidentiality	37	46.8
Ashamed of having TB	21	26.6
Lack of trust to future care for their TB	20	25.3
Fear of testing for TB	17	21.5
Pre- tending to be sick to get away from work	42	53.2
Others	4	5.1

N.B. Percent might not add up to 100 due to multiple responses. The cases are less than sample size and are different for different variables due to the differences in response

### Factors associated with Tuberculosis infection control practice

In the multivariate logistic regression, working department, knowing the presence of TBIC plan or not and knowing the presence of national guideline in the health center or not showed statistically significant association with TBIC practice whereas age, sex, site of health facility, marital status, religion, type of profession, educational level and working experience of the participants did not show statistically significant association with TBIC practice.

It was found that health workers who were working in TB clinic [AOR = 10.17, 95 % CI: 3.81–27.17], adult OPD [AOR = 3.23, 95 % CI: 1.54 - 6.79] and ART or inpatient [AOR = 2.74, 95 % CI: 1.13, 6.63] departments were more likely to practice TBIC than those who were working in pharmacy department.

It was also found that knowing either the presence of TBIC plan in health facility or not and TBIC practice has showed strong significant association. Health workers who knew the presence of TBIC plan in their health center or not were 4 times more likely to practice TBIC than those who didn’t knew, [AOR = 4.25, 95 % CI: 2.46 - 7.35]. It was also found out that health workers who knew either the presence of national guideline for TBIC in their health center or not were nearly 9 times more likely to practice TBIC than those who didn’t know it [AOR = 8.95, 95 % CI: 4.35,18.40] (Table 3).

**Table 3:** Bivariate and multivariate logistic regression analysis of factors associated with TBIC practice of health care workers in West Gojjam Zone, Northwest Ethiopia, August 2014

	TBIC practice			
Variables	Proper	Improper	COR 95 % CI	AOR 95 % CI
Site of health facility				
Urban	82	139	0.94 (0.67,1.32)	
Rural	158	252	1	
Sex				
Male	148	237	1.05 (0.75,1.45)	
Female	92	154	1	
Educational level				
Degree	79	78	1.97 (1.37,2.84)	1.20 (0.77,1.89)
Diploma	161	313	1	
Age category				
25–34 years	132	210	1.21 (0.86,1.70)	0.84 (0.56,1.26)
> 34 years	22	15	2.83 (1.40,5.74)	1.20(0.54,2.67)
18–24 years	86	166	1	1
Experience category				
3–5 years	65	80	1.76 (1.19,2.61)	1.11 (0.68,1.79)
5–10 years	29	26	2.42 (1.37,4.29)	1.73 (0.85,3.52)
> 10 years	23	18	2.77 (1.44,5.33)	1.05 (0.35,3.13)
1–3 years	123	267		1
Profession				
Health officer	44	37	6.34(2.98,13.51)	3.00(0.20,45.6)
Laboratory technologist	24	46	2.78(1.26,6.13)	1.56(0.13,19.36)
Midwifery	13	56	1.24(0.52,2.93)	2.29(0.15,35.58)
Nurse	147	188	4.17(2.17,8.01)	3.48(0.25,49.35)
pharmacy	12	64	1	1
Working department				
TB clinic	42	10	22.40(8.88,56.49)	10.17(3.81,27.17)*
Adult OPD	82	105	4.17(2.11,8.23)	3.23(1.54,6.79)*
Under 5 OPD	27	48	3.00(1.38,6.52)	2.02(0.87,4.69)
Laboratory	24	46	2.78(1.26,6.13)	1.71(0.73,4.00)
ART Clinic and IPD	24	34	3.77(1.68,8.45)	2.74(1.13,6.63)*
MCH room	29	84	1.84(0.87,3.89)	1.63(0.72,3.67)
Pharmacy	12	64	1	1
Training on TBIC				
Yes	122	96	3.18 (2.26,4.47)	1.30 (0.83,2.04)
No	118	295	1	1
Knowledge presence of TBIC plan				
Yes	221	239	7.40 (4.44,12.32)	4.25 (2.46,7.35)*
No	19	152	1	1
Know presence of national guideline for TBIC				
Yes	231	247	14.96 (7.45,30.04)	8.95 (4.35,18.40)*
No	9	144	1	1

* Statistically significant

## Discussion

Only 38 % of the study participants had proper overall TBIC practice in West Gojjam Zone. Measures of TBIC practices were variable from good in practicing natural ventilation (89.2 %) to low (23.5 %) use of N95 respirators by staff at the health centers.

Even though TBIC focal persons were assigned in each health centers the presence of TBIC policy/plan and national guideline for the management of TB was known by 72.9 and 75.8 % of participants respectively. This finding is in line with the study in South Africa [4] in which despite a TBIC officer appointed, there was no TBIC policy/plan or monitoring in place and it was not announced to all health workers. This may be due to the gap that health centers and woreda health offices didn’t give emphasis to the program and it was left to those health workers working in TB rooms.

In this study only 34.5 % of participants were ever trained on TBIC which is lower than the study done in Uganda (48.6 %) [17]. The difference may be due to the fact that the resources and budget allocated for the program were scarce and trainings were given by NGOs working in the area in collaboration with government to improve the TBIC program activities possibly leading to lower level of coverage.

The study found out that 71.6 % of health workers reported that they were doing TB screening at their department. This is higher than the study done in Uganda in which only 43 % reported screening patients for cough as they enter the facility [17]. The difference may be attributed to the fact that TBIC activities are currently getting attention and screening was done in all health care facilities because of the easy screening process that does not need a special technique and equipment.

Nearly two-thirds (60 %) of participants reported that they were ‘always’ informing coughing patients about cough hygiene in the quantitative part. This is lower than the study in South Africa (77.4 %) [4]. The difference may be due to lack of motivation and negative attitude towards TB among health workers that leads to low level of providing information on cough hygiene. The other reason may be the lack of adherence to preventive measures among patients which emerged as barrier to practicing TBIC activities in the qualitative part of the study. This is in agreement with the smaller number of health workers practicing on educating cough hygiene in the quantitative part.

Many HCWs had negative attitudes towards TBIC activities. HCWs were not interested in TB related work and therefore TBIC was left to the staff working in the TB clinics. Especially health workers working in pharmacy, MCH room, laboratory and inpatient departments didn’t give enough attention to TBIC practice. This may be due to lack of trainings and the already narrow chance of trainings on TBIC was given to those working in OPD and TB clinics. The other challenges mentioned included patients being non-compliant with cough hygiene. It was reported that where HCWs make an effort to implement the TBIC measures, patients don’t adhere to the instructions they are given.

Majority of participants (62.1 %) reported that they were giving priority for coughing patients which is higher than the study in Uganda in which coughing patients were not given priority in outpatients departments in over 90 % (47/51) of the facilities studied [17]. The deference may be due to the high level of attention given to the program in Ethiopia currently.

In this study majority (84.8 %) of study participants reported that they were comfortable to know their personal HIV status in their health facility and 54.4 % of who are comfortable with redeployment from high-risk areas if they are hypothetically HIV positive. This is lower than the findings from the study in South Africa [4]. The difference may be due to health workers afraid of disclosing themselves when changing their working department from high risk area to the lower one because of the perceived stigma from the other staff members.

Most (89.2 %) of the participants were always leaving doors and windows open in their working area to prevent the spread of TB and it is higher than the study in South Africa (69 %) [4]. The difference may be due to lack of options to environmental control measures in rural health centers other than natural ventilation in this study and the study in South Africa was conducted in hospital setting and there may be other alternatives that can be used as an option for environmental control measures than opening windows and doors. Natural weather conditions may be colder in South Africa than Ethiopian rural study area. All of the study participants used natural ventilation and there were no other alternatives like room air cleaners and UVGI in the upper room to prevent the spread of TB. The above findings are the same with the study done in China [18], in which mechanical ventilation was not available in any of the TB centers, and Nigeria [19] in which though 66.7 % of the consulting rooms were well ventilated, 25 % of them were overcrowded and no facility had air cleaners.

Only 12 % of those participants reported that there were respirators in their work rooms and from these 23 % of them ‘always’ used a respirator when in a room with a TB case/suspect. But from the total participants only 3 % were using respirators. The above finding is the same with the studies in Uganda in which no facility had N95 respirators for staff to use and China in which N95 respirators were not available for HCWs and surgical masks were not available for TB patients and suspects [17, 18]. This may be due to lack of financial resources to purchase the masks which was also supported by the qualitative interviews in which it was reported that lack of funds to buy masks and to carry out renovations and structural improvements as major challenges.

The overall level of proper TBIC practice in this study was 38 %. This finding is in line with the finding from a study done in Iraq in which only 38.2 % handled suspected TB cases correctly [20]. But it was lower than the finding in the study done in Ethiopia in which 63.3 % of participants had good TBIC status [21]. The difference with the previous Ethiopian study findings may be because the former Ethiopian study was conducted at hospital level and health workers at hospitals were more of senior and had more exposure to TBIC practice than those working in health centers in addition to better resources at hospital.

It was found that health workers working in TB clinics, adult OPD and ART or IPD departments were more likely to practice TBIC than those working in pharmacy department. This finding clearly revealed that the task of TBIC was left to those health workers working in OPDs and TB clinics. The finding in the qualitative key informant interview and observation also showed that HCWs had negative attitude towards TB treatment and control activities. It was expressed that many HCWs were not interested in TB related work and therefore TBIC control is left to the staff working in the TB clinics. Especially health workers working in pharmacy, maternal and child health, laboratory and inpatient departments didn’t give attention to TBIC practice. This may be because these groups of professionals were not oriented and motivated to do TBIC activities and thus became ignorant and careless to perform TBIC activities.

This study showed that knowing the presence of TBIC plan in health facility was independent predictor of TBIC practice. Health workers who knew whether TBIC plan is present at the health center or not were more likely to practice TBIC activities (AOR = 4.25). This might be due to the fact that knowing what was planned was the base to practice accordingly. After adjusting for confounding factors it was also seen that health workers who knew the presence of national guideline in their health facility were more likely practiced TBIC than those who do not know(AOR =8.95). This might be due to the reason that those who know the presence of national guideline were using it for their daily TBIC activities.

Majority of participants endorsed a number of barriers to better implementation of TBIC activities. Commonly cited barriers includes lack of training and knowledge, lack of infrastructures and resources, lack of supplies and man power, lack of awareness to the community and carelessness and lack of commitment to HCWs. Majority of participants cited stigma or confidentiality concerns as barriers to staff TB/HIV testing and redeployment of HIV-positive HCWs from high risk areas. These findings are in line with a review finding from similar studies in South Africa [4, 22], Uganda [17], China [18], Nigeria [19], Russia [23] and Malawi [24].

This study showed that poor TBIC practice characterizes HCWs working in all departments other than those working in TB clinics among HCWs in health centers. This clearly revealed the urgency to implement initiatives for improving TBIC activities. It can be used to inform that the design and implementation of a feasible and successful facility TBIC program at primary health care unit level. These findings provide more specific details regarding TBIC implementation in rural primary health care and this report provides new information that could be applied to similar institutional settings. The assessment of staff TBIC practice provides useful baseline information regarding deficits and barriers to effective TBIC practice and strategies. This approach can demonstrate a facility’s strengths and weaknesses, highlight pertinent HCWs concerns, and determine where to concentrate efforts.

## Conclusions

The overall proper TBIC practice by health workers in health centers of West Gojjam Zone was low. The necessary settings and supplies were not available in majority of health centers. Tuberculosis infection control practices of health care workers was determined by their working area/department, knowledge status of TBIC plan and presence of national TBIC guide line in their health centers.

Health centers should prepare TBIC plans, orient all health workers and should monitor the activity accordingly. Integrated supportive supervision by the Woreda Health Offices should make efforts to give orientation and clarity on TBIC plan and the proper implementation of TBIC activities. There is a need to fulfill the necessary supplies and setting for TBIC activities at the health centers. Training on TBIC should be given to all health workers with emphasis to those working in maternal and child health, pharmacy and laboratory departments to enhance the TBIC practice and prevent spread of TB at the health facilities.

## Abbreviations

DOTS, directly observed treatment short-course; HCW, health care worker; IPD, Inpatient Department; LMICs, low- and middle-income countries; LTBI, latent tuberculosis infection; MDRTB, multi-drug resistant tuberculosis; OPD, Outpatient Department; PPE, personal protective equipment; TBIC, tuberculosis infection control; UVGI, ultraviolet germicidal irradiation; X-DRTB, extensively drug resistant tuberculosis
